# Significance of lncRNA CDKN2B-AS1 in Interventional Therapy of Liver Cancer and the Mechanism under Its Participation in Tumour Cell Growth via miR-199a-5p

**DOI:** 10.1155/2022/2313416

**Published:** 2022-08-30

**Authors:** Lu Xu, Haotian Wu, Jianhua Pan, Zhiheng Chen, Linan Du

**Affiliations:** Department of Interventional Ultrasound, The Second Affiliated Hospital of Anhui Medical University, Hefei, Anhui 230000, China

## Abstract

**Methods:**

Totally 34 LC patients admitted to our hospital between January 2020 and March 2021 (Obs group) and 32 healthy individuals over the same time span (Con group) were enrolled. CDKN2B-AS1 and miR-199a-5p in the two groups were PCR quantified, and their association and value for the diagnosis and therapy of LC were analyzed. In addition, purchased LC cells were adopted for *in vitro* assays, and the influences of CDKN2B-AS1 and miR-199a-5p on biological behaviours of LC cells were assessed through CCK-8, Transwell, and flow cytometry experiment, and their regulatory association was verified by the dual luciferase reporter (DLR) assay and rescue assay. And the autophagic protein expression was tested by the western blot to confirm the effect of both on the autophagic capacity of LC cells.

**Results:**

CDKN2B-AS1 in LC cases presented high expression and dropped after therapy (*P* < 0.05), and the opposite situation of miR-199a-5p was found in the LC cases (*P* < 0.05). *In vitro* assays, after silencing of CDKN2B-AS1 and upregulation of miR-199a-5p, LC cells presented weaker viability, invasion and migration activities, and stronger apoptotic activity (all *P* < 0.05). The DLR assay revealed suppressed fluorescence activity of CDKN2B-AS1-WT by miR-199a-5p (*P* < 0.05). Moreover, according to the rescue assay, the impacts of silencing CDKN2B-AS1 on LC cells could be completely offset by silencing miR-199a-5p (*P* < 0.05). According to the clone formation and WB assay, the growth and autophagy of LC cells were under the regulation of CDKN2B-AS1 targeting miR-199a-5p (*P* < 0.05).

**Conclusion:**

With high expression in LC cases, CDKN2B-AS1 is implicated in the development and progression of LC by suppressing cell autophagy through targeting miR-199a-5p.

## 1. Introduction

Liver cancer (LC) is one frequently seen and high-risk malignancy worldwide. Global tumour epidemiological statistics report over 800,000 new cases of LC worldwide in 2015, including over 50% of them in China [1,2]. Moreover, approximately 90% of LC patients die each year, and mortality ranks at the top among all tumours [3,4]. One primary cause of unfavourable prognosis of LC is that the absence of full clarification on its clinical pathogenic mechanism hinders the effective, rapid, and timely screening for LC without special clinical symptoms in the early phase of onset [5]. The advancement and application of targeted therapy, radiotherapy, and chemotherapy are beneficial to the prognosis of LC patients, but the drug resistance of the latter two in long-run treatment is still an urgent crucial problem [6,7]. In addition, recurrence of diseases is extremely frequently seen in patients after radiotherapy and chemotherapy [8]. All the above limitations urgently entail a novel potential diagnosis and therapy scheme of LC to improve patients' prognoses.

As one hot research direction over the past few years, long-chain noncoding RNA (lncRNA) serves as a crucial part in regulating the development of various tumours [9]. Among them, CDKN2B-AS1 in the *CDKN2B-CDKN2A* gene cluster of human chromosome 9p2 was firstly found to have an abnormal expression in diseases such as periodontitis and endometriosis [10]. As the research deepens, CDKN2B-AS1 has been indicated to be crucial in the liver as well. For instance, Xiao et al. have pointed out its promotion of the development of liver fibrosis [11,12]. Burd et al. have revealed that CDKN2B-AS1 can interact with PRC1 and PRC2 and thus give rise to epigenetic silencing of other genes in this cluster [13], and both PRC1 and PRC2 have been verified to be crucial in hepatitis and LC [14,15]. Zhuang et al. have also found that CDKN2B-AS1 was one lncRNA with abnormal expression in LC during screening [16]. Not only that, Zhuang et al. and Song et al. have confirmed that CDKN2B-AS1 has been found to be an independent prognostic factor for LC and promotes the growth of LC cells through let-7c-5p/NAP1L1 axis [16, 17], thus showing the importance of CDKN2B-AS1 for LC. However, all the current studies have certain limitations. For example, the detection of CDKN2B-AS1 by Zhuang et al. was performed only through LC tissues, and the expression of CDKN2B-AS1 in the blood and the changes during pathological changes in LC remain vague. In contrast, Song et al. have not yet validated the pathway of action of CDKN2B-AS1, and they also suggested that the effect of CDKN2B-AS1 on LC cells may not only be through the let-7c-5p/NAP1L1 axis.

Currently, the implication of CDKN2B-AS1 in the development of tumours by regulating downstream target genes has been confirmed by many studies [18, 19]. To understand the action mechanism of CDKN2B-AS1 on LC, its downstream target genes were forecasted through an online database, and its targeted regulatory relationship with miR-199a-5p was found [20]. Prior research has also pointed out the possibly great significance of miR-199a-5p to LC [21]. Therefore, we suspect that CDKN2B-AS1 may have an effect on LC through miR-199a-5p, but studies are lacking to confirm it.

For the purpose of learning more about the impacts of CDKN2B-AS1 on LC and its clinical value, we analyzed CDKN2B-AS1 expression in LC cases, its changes during interventional therapy, and the mechanism of action, with the goal of offering a novel reference for the future diagnosis and therapy of LC and laying a reliable foundation for the follow-up research.

## 2. Materials and Methods

### 2.1. Patients' Data

Totally 34 LC patients admitted to our hospital between January 2020 and March 2021 (Obs group) and 32 healthy individuals over the same time span (Con group) were enrolled and prospectively analyzed. All patients met the following conditions: confirmed with primary LC by biopsy, without invasion and metastasis, in early pathological stage, had not received anti-tumour therapy before admission, cooperated with follow-up, with estimated survival >1 month All the subjects signed the informed consent form by themselves.

### 2.2. Treatment means

After admission, patients were treated with TAE/TACE intervention according to indications. After the operation, there was no need for fasting routinely, and tumours in adjacent cavity organs were ablated. The fasting time of foods and water was adjusted according to specific conditions. The patients' vital signs were monitored for 24 h, and they were required to stay in bed for over 12 h. Their blood routine and liver and kidney function were also monitored. One course spanned 30 days. Each patient was treated with each course once, a total of 3 consecutive courses. Clinical efficacy evaluation [22]: Complete remission (CR): the lesion completely disappears for over 4 weeks; partial remission (PR): the product of the maximum diameter of the tumour and the maximum diameter is reduced by over 50%; stable disease (SD): the product of them is reduced by more than 25% but less than 50%; and progressive disease (PD): the lesion has no alleviation.

### 2.3. Sample Collection

Fasting peripheral blood (5 mL) was acquired from each patient before therapy (T0), after 1 course of therapy (T1), 2 courses of therapy (T2), and 3 courses of therapy (T3), and from each healthy individual at admission, followed by centrifugation to acquire serum that was then saved in a refrigerator at −80°C for later analyses.

### 2.4. Cell Origin and Transfection

Human LC cell SMMC-7721, Huh7, and normal liver epithelial cell HL‐7702 all from ATCC were treated with incubation (37°C) in DMEM (10% FBS and 1% penicillin-streptomycin) under 5% CO_2_. si-CDKN2B-AS1, miR-199a-5p mimics (miR-mimics), miR-199a-5p inhibition (miR-inhibition), and corresponding blank vectors (NC-CDKN2B-AS1, miR-NC) all offered by GenScript Biotechnology Co. Ltd. were transfected into different LC cells under the guidelines of a Lipofectamine 2000 kit.

### 2.5. qRT-PCR Assay

Total RNA acquired from samples via Trizol was treated with reverse transcription via a PrimeScript RT kit (Takara, Japan), followed by PCR amplification under the guidelines of Thunderbird SYBR®qPCR Mix (Takara, Japan). 2^−ΔΔCT^ was adopted for relative expression calculation (internal reference: GAPDH and U6), and the primer sequences are shown in Table 1.

### 2.6. CCK-8 Assay

Resuspended cells were transferred to a 96-pore plate (8 × 10^3^ cells/pore). After they were attached to the wall and sprawled out, DMEM was put in for 24 h incubation, followed by discarding of the supernatant. At 24, 48, and 72 h after incubation, 10 *μ*L CCK-8 solution was put into each pore, followed by the determination of optical density (450 nm) via one microplate reader.

### 2.7. Transwell Assay

Invasion ability detection: cells (1 × 10^5^ cells/mL) and 500 *µ*L medium (no serum) were transferred to the upper and lower compartments of the Transwell chamber, respectively. After 24 h incubation, the membrane-penetrating cells were treated by immobilization, staining, and microscopical examination. Migration ability test: add 50 *μ*L of Mataigel gel diluent to the upper compartment, and the others are the same as above.

### 2.8. Flow Cytometry

Transfected cells were trypsinized, followed by twice washing via PBS and then measurement of apoptotic activity via an Annexin V-FITC kit. Lastly, a flow cytometer was adopted for result analysis.

### 2.9. Target Gene Forecasting and Dual Luciferase (DL) Reporter Assay

Based on the online target gene forecasting website, target genes of miR-199a-5p were forecasted, and miR-199a-5p binding loci-containedwild-type (WT) fragment of CDKN2B-AS1 and its mutant fragment (MUT) were inserted into the downstream of pmirGLO DL expression vectors, respectively, followed by transfection with miR-mimics and then 48 h incubation. Lastly, the luciferase activity was measured via the system.

### 2.10. Western Blot (WB) Assay

Cells were lysed via RIPA, followed by SDS-PAGE, and then transferred to a membrane that was then treated with immersion in 5% defatted milk and overnight incubation (4°C) with I antibodies Beclin-1, LC3I/II, as well as *β*-atin. After washing, the membrane was treated with 2 h incubation (indoor temperature) with anti-IgG coupled with HRP, followed by ECL development and analysis of gray value via ImageJ software.

### 2.11. Rescue Assay

LC cells were cotreated with si-CDKN2B-AS1 and miR-inhibition, and some cells were transfected with si-CDKN2B-AS1 or NC-CDKN2B-AS1. The biological behaviours of all cells were detected with the above methods. Additionally, cells at the log growth stage were transferred to a 6-pore plate, and the medium was replaced with a fresh one once the cells were attached to the wall and sprawled out. When approximately 50 clustered communities could be observed, the supernatant was discarded, and the cells were treated by washing via PBS, immobilization via methanol, and staining via Giemsa. Finally, the number of clones was counted based on the photographed figure.

### 2.12. Statistical Analyses

This study adopted SPSS22.0 for data analyses. Intergroup comparisons of measurement data (mean ± SD) were performed via the independent-samples*T* test, and multigroup comparisons of them were performed via the one-way ANOVA and LSD-t post hoc test. Intergroup comparisons of counting data were carried out via the chi-square test, and Pearson's correlation coefficient was adopted for correlation analyses. ROC curves were adopted for the analysis of predictive value. *P* < 0.05 implies a notable difference.

## 3. Result

### 3.1. CDKN2B-AS1 and miR-199a-5p in LC

**Figure 1:**
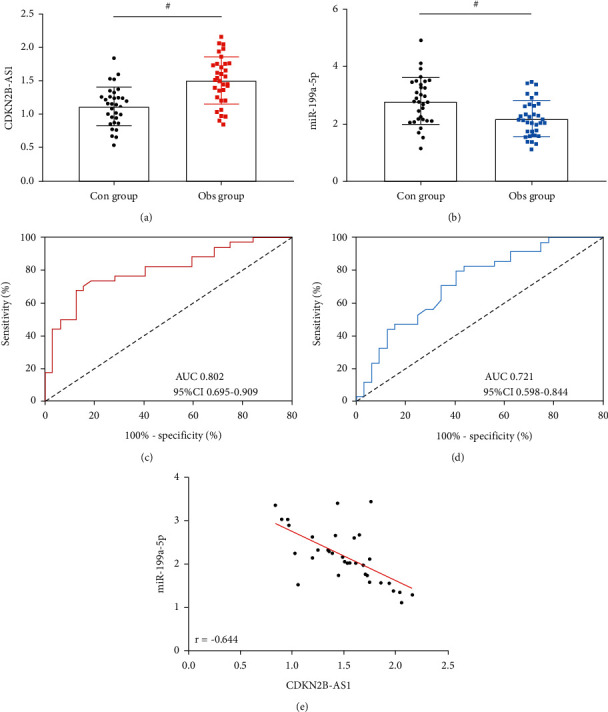
Expression of CDKN2B-AS1 and miR-199a-5p in LC: (a) CDKN2B-AS1 expression at T0, (b) MiR-199a-5p expression at T0, (c) ROC curve of CDKN2B-AS1 for forecasting the occurrence of LC, (d) ROC curve of miR-199a-5p for forecasting the occurrence of LC, and (e) association of CDKN2B-AS1 with miR-199a-5p in expression. “^#^” means there is a statistical difference between the groups (*P* < 0.05).

The determination results revealed a higher peripheral blood CDKN2B-AS1 level (Figure 1(a)) and a lower miR-199a-5p level (Figure 1(b)) in the Obs group than in the Con group at T0 (both *P* < 0.05). According to ROC curve-based analysis, CDKN2B-AS1 > 1.38 had a sensitivity and specificity of 87.50% and 79.41%, respectively, in forecasting the occurrence of LC (*P* < 0.001; Figure 1(c)), and miR-199a-5p < 2.68 had a sensitivity and specificity of 79.41% and 59.38%, respectively, in forecasting it (*P* < 0.001; Figure 1(d)). Pearson's correlation coefficient analysis revealed a negative association of CDKN2B-AS1 with miR-199a-5p in peripheral blood in terms of expression at T0 (*r* = −0.644, *P* < 0.05; Figure 1(e)).

### 3.2. Value of CDKN2B-AS1 and miR-199a-5p in Interventional Therapy of LC

**Figure 2:**
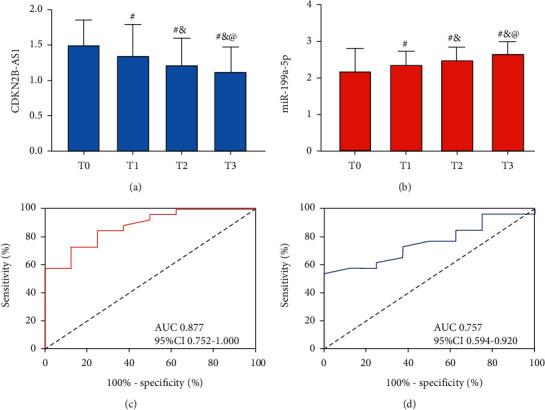
Value of CDKN2B-AS1 and miR-199a-5p in interventional therapy of LC: (a) changes in CDKN2B-AS1 expression during therapy, (b) changes in miR-199a-5p expression during therapy, (c) ROC curve of CDKN2B-AS1 at T2 in forecasting clinical efficacy, and (d) ROC curve of miR-199a-5p at T2 in forecasting clinical efficacy. “^#^” means there is a difference compared with T0; “&” means there is a difference compared with T1; and “@” means there is a difference compared with T2 (*P* < 0.05).

Peripheral blood CDKN2B-AS1 in the Obs group dropped in a time-dependent manner, namely, the highest at T0 and the lowest at T3 (*P* < 0.05; Figure 2(a)), and peripheral blood miR-199a-5p in the group elevated in such a manner (*P* < 0.05; Figure 2(b)). The patients were assigned to a favourable-efficacy group (with clinical efficacy of CR or PR) and a good-efficacy group (with clinical efficacy of SD and PD) in light of the clinical efficacy. Their CDKN2B-AS1 and miR-199a-5p at T2 were evaluated using ROC curves. According to evaluation results, CDKN2B-AS1 < 1.26 at T2 had a sensitivity and specificity of 73.08% and 87.50%, respectively, in forecasting favourable efficacy (*P* < 0.001; Figure 2(c)) and miR-199a-5p > 2.49 at T2 a sensitivity and specificity of 53.85% and 100.0%, respectively, in forecasting it (*P* < 0.001; Figure 2(d)).

### 3.3. Online Database Analysis of the Expression of CDKN2B-AS1 and miR-199a-5p

**Figure 3:**
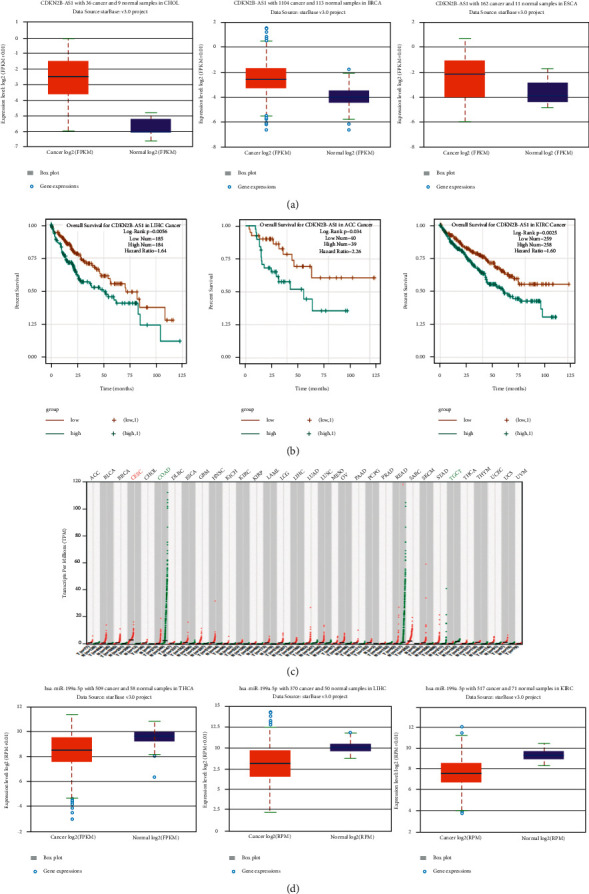
Online database analysis of the expression of CDKN2B-AS1 and miR-199a-5p: (a) the ENCORI database analyzes the expression of CDKN2B-AS1, (b) the ENCORI database analyzes the relationship between CDKN2B-AS1 and the prognosis of tumour diseases, (c) GEPIA database analyzes the expression of CDKN2B-AS1, and (d) ENCORI database analyzes the expression of miR-199a-5p.

In the ENCORI database, we found that CDKN2B-AS1 is also highly expressed in cholangiocarcinoma, breast cancer, and esophageal cancer (Figure 3(a)). Moreover, through prognostic analysis of tumour diseases, we found that patients with high expression of CDKN2B-AS1 in LC, adrenocortical carcinoma, and renal clear cell carcinoma generally have a lower prognostic survival rate (Figure 3(b)). Subsequently, CDKN2B-AS1 was further screened in the GEPIA database, and it was found that CDKN2B-AS1 was also highly expressed in LC (Figure 3(c)). In the screening of miR-199a-5p, we found that the expression of miR-199a-5p was also low in the thyroid, LC, and renal clear cell carcinomas (Figure 3(d)). However, in the analysis of miR-199a-5p and the prognosis of tumour diseases in the database, there is no difference due to the small sample size.

### 3.4. Mechanism under Impacts of CDKN2B-AS1 on LC Cells

**Figure 4:**
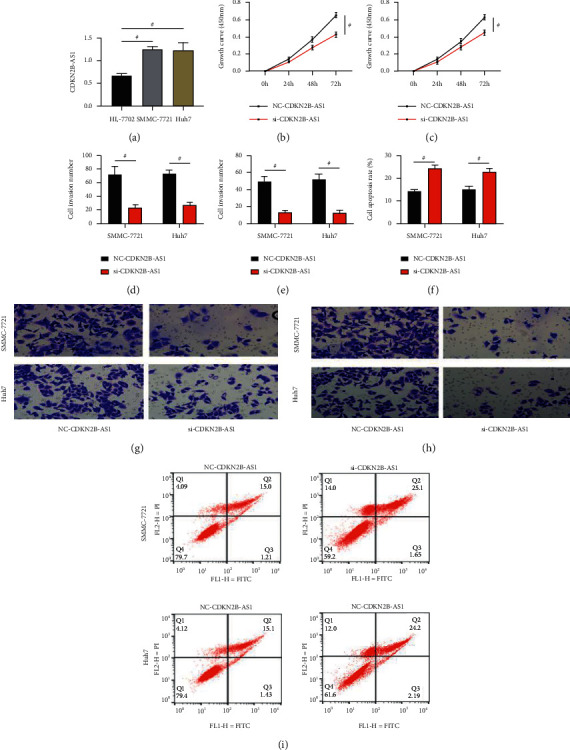
Mechanism under the impacts of CDKN2B-AS1 on LC cells (*n* = 3): (a) CDKN2B-AS1 expression in LC cells and normal liver cells, (b) impacts of CDKN2B-AS1 on the proliferation of SMMC-7721 cells, (c) impacts of CDKN2B-AS1 on the proliferation of Huh7 cells, (d) the number of invasive cells, (e) the number of migrate cells, (f) apoptosis rate, (g) staining of transmembrane cells, (h) staining of migrating cells, and (i) flow cytometry. “^#^” means there is a statistical difference between the groups (*P* < 0.05).

A higher CDKN2B-AS1 level was found in SMMC-7721 and Huh7 cells than in HL‐7702 cells (*P* < 0.05; Figure 4(a)), which verified the above experimental results. The CCK-8 assay revealed notably weaker proliferation activity in the si-CDKN2B-AS1 group than in the NC-CDKN2B-AS1 group (*P* < 0.05; Figures 4(b) and 4(c)). The Transwell assay revealed notably weaker invasion activities in the si-CDKN2B-AS1 group than in the NC-CDKN2B-AS1 group (*P* < 0.05; Figures 4(d) and 4(e)). Not only that, but the cell migration number of the si-CDKN2B-AS1 group was also significantly lower than that of the NC-CDKN2B-AS1 group (*P* < 0.05; Figures 4(f) and 4(g)). The flow cytometry revealed stronger apoptotic activity in the si-CDKN2B-AS1 group than in the NC-CDKN2B-AS1 group (*P* < 0.05; Figures 4(h) and 4(i)). It follows from the above results that silencing CDKN2B-AS1 can enhance the apoptotic activity of LC cells and suppress their viability.

### 3.5. Mechanism under Impacts of miR-199a-5p on LC Cells

**Figure 5:**
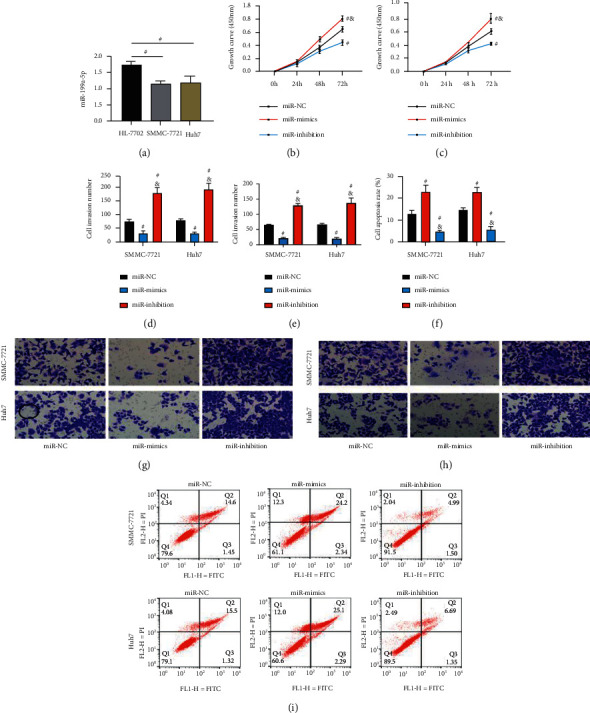
Mechanism under the impacts of miR-199a-5p on LC cells (*n* = 3): (a) miR-199a-5p expression in LC cells and normal liver cells, (b) impacts of miR-199a-5p on the proliferation of SMMC-7721 cells, (c) impacts of miR-199a-5p on the proliferation of Huh7 cells, (d) the number of invasive cells, (e) the number of migrate cells, (f) apoptosis rate, (g) staining of transmembrane cells, (h) staining of migrating cells, and (i) flow cytometry. “^#^” means there is a difference compared with the miR-NC group, and “&” means there is a difference compared with the miR-mimics group (*P* < 0.05).

Similarly, quantification of miR-199a-5p via PCR revealed lower miR-199a-5p expression in SMMC-7721 and Huh7 cells than in HL‐7702 cells (*P* < 0.05; Figure 5(a)). According to the CCK-8 assay, after transfection with miR-199a-5p mimics and inhibitor sequence, the miR-mimics group presented weaker proliferation activity than the miR-inhibition and miR-NC groups, and the miR-inhibition group presented stronger proliferation activity than the miR-NC group (all *P* < 0.05; Figures 5(b) and 5(c)). The Transwell assay revealed weaker cell invasion activities in the miR-mimics group than in the other two groups and stronger activities of them in the miR-inhibition group than in the miR-NC group (*P* < 0.05; Figures 5(d) and 5(e)). Similarly, we found that the cell migration number of the miR-mimics group was the lowest among the three groups, while the migration number of the miR-inhibition group was significantly higher than that of the miR-NC group (*P* < 0.05; Figures 5(f) and 5(g)). According to the flow cytometry, the highest apoptosis rate was found in the miR-mimics group, followed by the miR-NC group and the miR-inhibition group from high to low (*P* < 0.05; Figures 5(h) and 5(i)). It follows from the above results that silencing miR-199a-5p can enhance the viability of LC cells while upregulating it can suppress the viability.

### 3.6. Association of CDKN2B-As1 with miR-199a-5p

**Figure 6:**
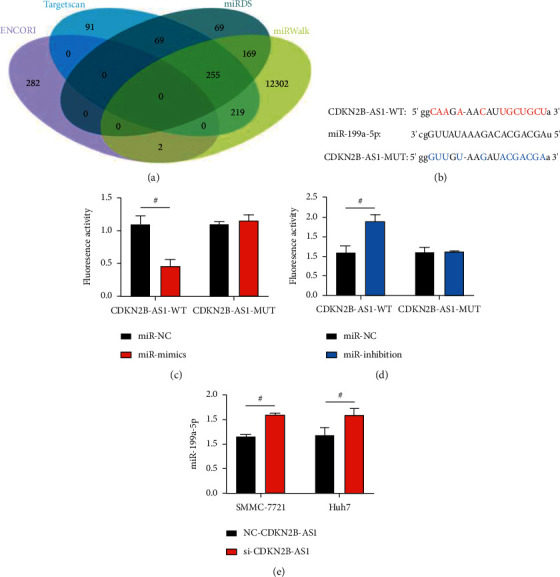
Association of CDKN2B-AS1 with miR-199a-5p (*n* = 3): (a) association analysis of CDKN2B-AS1 with miR-199a-5p based on online target gene forecasting website ENCORI, Targetscan, miRDB, and miRWalk; (b) complementary binding loci of CDKN2B-AS1 with miR-199a-5p; (c) and (d) DL reporter assay; and (e) impacts of CDKN2B-AS1 on miR-199a-5p. “^#^” means there is a statistical difference between the groups (*P* < 0.05).

With the aim of confirming the association of CDKN2B-AS1 with miR-199a-5p, the downstream target genes of CDKN2B-AS1 were screened through the online target gene forecasting website (ENCORI, miRDB, and miRWalk), and complementary binding loci were discovered (Figure 6(a)); the site binding between the two is shown in Figure 6(b). The DL reporter assay revealed notably suppressed fluorescence activity of CDKN2B-AS1-WT after transfection of miR-mimics (*P* < 0.05; Figure 6(c)), and notably enhanced fluorescence activity of it after transfection with miR-inhibition (*P* < 0.05; Figure 6(d)), suggesting the target regulation of CDKN2B-AS1 on miR-199a-5p. Moreover, qualification of miR-199a-5p in LC cells treated with si-CDKN2B-AS1 revealed notably higher miR-199a-5p expression in the si-CDKN2B-AS1 group than in the NC-CDKN2B-AS1 group (*P* < 0.05; Figure 6(e)) suggesting the targeted suppression of CDKN2B-AS1 on miR-199a-5p.

### 3.7. Rescue Experiment

**Figure 7:**
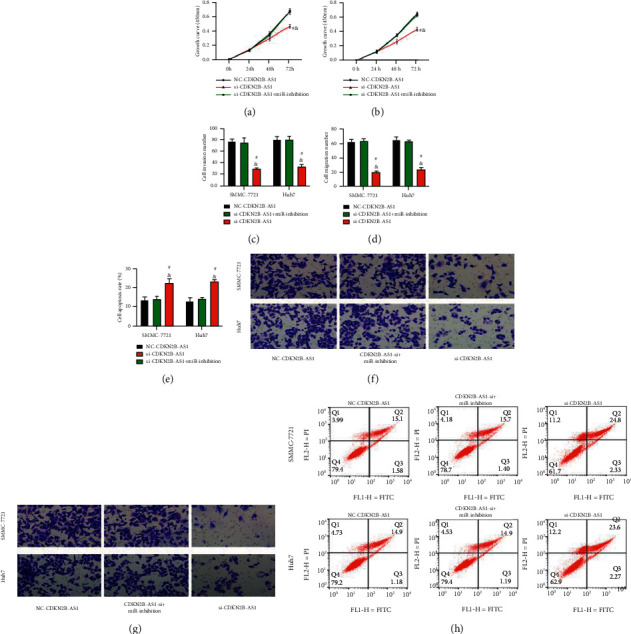
Rescue experiment: (a) growth curve of SMMC-7721 cells (*n* = 3), (b) growth curve of Huh7 cells, (c) the number of invasive cells, (d) the number of migrate cells, (e) apoptosis rate, (f) staining of transmembrane cells, (g) staining of migrating cells, and (h) flow cytometry. “^#^” means there is a difference compared with the NC-CDKN2B-AS1 group, and “&” means there is a difference compared with the si-CDKN2B-AS1 + miR-inhibition group (*P* < 0.05).

The si-CDKN2B-AS1 and miR-inhibition were transfected into LC cells together. In addition, the si-CDKN2B-AS1 group and the NC-CDKN2B-AS1 group that were transfected separately were set up for rescue experiments. In the CCK-8 experiment, we found that the cell proliferation ability of the si-CDKN2B-AS1 + miR-inhibition group was not different from that of the NC-CDKN2B-AS1 group (*P* > 0.05), which was significantly higher than that of the si-CDKN2B-AS1 group (Figures 7(a) and 7(b)). The Transwell experiment showed that the number of cell invasions in the si-CDKN2B-AS1 + miR-inhibition group and the NC-CDKN2B-AS1 group was similar (*P* > 0.05) and was higher than that in the si-CDKN2B-AS1 group (*P* < 0.05; Figures 7(c) and 7(d)). Similarly, the cell migration number of the si-CDKN2B-AS1 + miR-inhibition group and the NC-CDKN2B-AS1 group was not different (*P* > 0.05), which was higher than that of the si-CDKN2B-AS1 group (*P* < 0.05; Figures 7(e) and 7(f)). Finally, the results of flow cytometry showed that there was no difference in the apoptotic rate between the two groups (*P* > 0.05), which was significantly higher than that of the si-CDKN2B-AS1 group (*P* < 0.05; Figures 7(g) and 7(h)). It can be seen that the effect of inhibiting CDKN2B-AS1 on LC cells was completely reversed after inhibiting miR-199a-5p, confirming the targeted regulation relationship between the two.

### 3.8. Mediation of CDKN2B-AS1 to the Autophagy of LC Cells via miR-199a-5p

**Figure 8:**
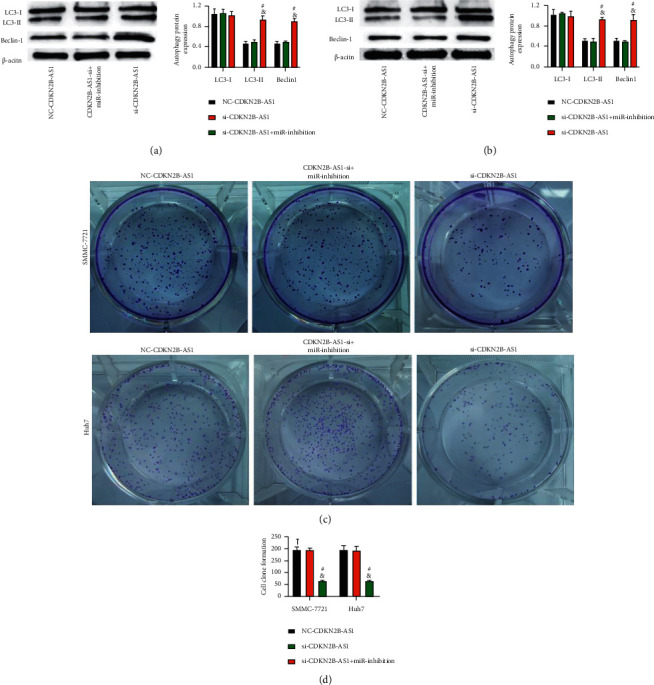
Mediation of CDKN2B-AS1 to the autophagy of LC cells via miR-199a-5p (*n* = 3): (a) autophagy protein detection results in SMMC-7721 cells, (b) autophagy protein detection results in Huh7 cells, (c) cell clone formation experiment, and (d) the number of cell clones formed. “^#^” means there is a difference compared with the NC-CDKN2B-AS1 group, and “&” means there is a difference compared with the si-CDKN2B-AS1 + miR-inhibition group (*P* < 0.05).

The WB assay revealed higher expression of LC3-II and Beclin-1 in the si-CDKN2B-AS1 group than in the NC-CDKN2B-AS1 group (*P* < 0.05) and similar expression of them between the si-CDKN2B-AS1 + miR-inhibition and NC-CDKN2B-AS1 groups (*P* > 0.05; Figures 8(a)–8(d)). The clone formation experiment showed that the number of cell clones in the NC-CDKN2B-AS1 group and the si-CDKN2B-AS1 + miR-inhibition group was similar, and both were higher than that in the si-CDKN2B-AS1 group (*P* < 0.05; Figures 8(e) and 8(f)). The results imply the function of suppressing CDKN2B-AS1 to enhance the autophagy of LC cells and decrease the synthesis ability of cells and the ability of miR-inhibition to reverse the impacts of si-CDKN2B-AS1 on autophagy.

## 4. Discussion

LC is a frequently seen clinical malignancy, with high rates of metastasis and recurrence that directly give rise to unsatisfactory prognosis and low survival rate [23]. Current research has revealed the involvement of lncRNA in the development of LC. Our study has revealed the potential of CDKN2B-AS1 to be a diagnostic index of LC because of its aberrant expression in peripheral blood and cells of LC cases and has also demonstrated that it is probably a therapeutic target for its regulation of the biological behaviours of LC cells. For LC with increasing morbidity and mortality, this may be a breakthrough in clinically conquering LC. This can provide patients with a more reliable prognosis and reduce the potential threat of LC.

Prior research has reported the differential expression of CDKN2B-AS1 in diseases including renal clear cell carcinoma [24], lung cancer [25], and ovarian cancer [26], but its association with LC is rarely studied. Similar to the expression analysis of CDKN2B-AS1 in TCGA, peripheral blood CDKN2B-AS1 increased in LC patients in our study, which suggested the possible involvement of CDKN2B-AS1 in the development of LC. The expression result is also in agreement with prior research [27, 28], which verifies the accuracy of our study. Moreover, ROC curve-based analysis also demonstrated the excellent performance of CDKN2B-AS1 in forecasting the occurrence of LC, which preliminarily suggested the potential of CDKN2B-AS1 to one marker of LC. Interventional therapy, as the first choice for noninvasive therapy of LC at the current stage, is frequently adopted in clinical practice [29]. During interventional therapy, there is an absence of a fast and effective detection means to judge the changes in patients' condition and to assist the clinic to adjust interventional therapy in time. Based on the above results, we through CDKN2B-AS1 might have this potential. Accordingly, the follow-up determination revealed that CDKN2B-AS1 gradually dropped during the interventional therapy and demonstrated excellent prediction performance in clinical efficacy on patients. It verifies the importance of CDKN2B-AS1 in LC again and also suggests the feasibility of evaluating the changes in patients' conditions via CDKN2B-AS1 in the future clinic. In our *in vitro* assay, after silencing of CDKN2B-AS1, LC cells presented substantially enhanced apoptotic activity and substantially suppressed invasion, proliferation, as well as migration, suggesting the oncogene role of overexpressed CDKN2B-AS1 in LC and its potential to be the therapeutic target. Not only that, but in the studies of Dasgupta et al. and Luo et al., CDKN2B-AS1 is also considered to be a potential marker for renal cancer and osteosarcoma [24, 30], which also shows the significance of CDKN2B-AS1 on tumour diseases and suggests that we will be able to do so in the future. CDKN2B-AS1 and even more LncRNAs may be the key to conquering tumours.

MiR-199a-5p in LC patients was quantified, and analyzed, which revealed its low expression in LC, its increase during interventional therapy, and its excellent performance in forecasting the occurrence of and clinical efficacy on LC. *In vitro* assay revealed that silencing miR-199a-5p expression could weaken the apoptotic activity of LC cells and accelerate their invasion, proliferation, and migration while increasing it could give rise to opposite results, which also confirmed the impacts of miR-199a-5p on LC. Furthermore, the correlation analysis revealed a negative association between CDKN2B-AS1 and miR-199a-5p in terms of expression, and the DL reporter assay verified a targeted regulation association between them. The results revealed the participation of CDKN2B-AS1 in the development of LC through targeted inhibition on miR-199a-5p.

Finally, according to the rescue assay, inhibiting CDKN2B-AS1 expression can enhance the autophagy ability of LC cells and also greatly slow down their growth ability, while suppression of miR-199a-5p expression can completely reverse the impacts of CDKN2B-AS1 on the cell migration, invasion, proliferation, apoptotic activity, and autophagy ability. This verifies the targeted regulatory association of CDKN2B-AS1 with miR-199a-5p again and also implies that we can treat LC by suppressing CDKN2B-AS1 and improving the autophagy of LC cells in the future.

By combining the results of this experiment with previous related studies [27, 31, 32], we believe that CDKN2B-AS1 and miR-199a-5p can be used as clinical routine screening indicators, so as to improve the early diagnosis rate of LC and provide relevant treatment for patients in a timely manner. What is more, CDKN2B-AS1 with miR-199a-5p can be used as an indicator of disease progression in LC to aid clinical understanding of disease progression. Furthermore, after perfecting gene targeting therapy, CDKN2B-AS1 and miR-199a-5p can be used as targets to start targeted therapy, which may achieve better results and safety than current surgery and radiotherapy for tumours and provide more reliable life safety for patients.

Of course, this research also still has some limitations. In the first place, the number of patients included was small, and they were all early-stage LC, and the results for CDKN2B-AS1 with miR-199a-5p may be not all-inclusive. In the second place, the prognostic significance of CDKN2B-AS1 versus miR-199a-5p cannot be assessed at this time because prognostic follow-up was not performed. In the third place, due to limited medical record collection, we only obtained blood samples from LC patients for testing, and subsequent tissue samples of LC need to be obtained for validation analysis. Hence, we need to conduct more in vitro experiments to analyze and validate the mechanism of action of CDKN2B-AS1 and miR-199a-5p on LC and confirm the effect of both on actual tumour growth by live animal tumour transplantation.

## 5. Conclusions

With high expression in LC cases, CDKN2B-AS1 possesses excellent predictive performance on the occurrence of LC and the efficacy of interventional therapy. It can weaken the apoptotic activity and autophagy of LC cells and enhance their invasion, proliferation, and migration through targeted inhibition on miR-199a-5p and thus affect the development of LC, so it is promising to be a breakthrough in future diagnosis and therapy.

## Figures and Tables

**Table 1:** Primer sequences.

	*F*	*R*
CDKN2B-AS1	5′-TCATCATCATCATCATCATC-3′	5′-TGCTTCTGTCTCTTCATA-3′
*β*-actin	5′-CCTGGCACCCAGCACAAT-3′	5′-GGGCCGGACTCGTCATAC-3′
miR-199a-5p	5′-TCAAGAGCAATAACGAAAAATGT-3′	5′-GCTGTCAACGATACGCTACGT-3′
U6	5′-ATTGGAACGATACAGAGAAGATT-3′	5′-GGAACGCTTCACGAATTTG-3′

## Data Availability

The data sets used and analyzed in the current research are available from the corresponding author upon request.

## Additional Points

The aim of this research was to investigate the changes of CDKN2B-AS1 and miR-199a-5p during liver cancer (LC) intervention and the mechanisms of their effects on LC cells. We found that CDKN2B-AS1 was highly expressed in LC and decreased during the intervention, while miR-199a-5p was lowly expressed and increased. *In vitro* experiments manifested that silencing CDKN2B-AS1 inhibited the activity of LC cells and promoted apoptosis. Whereas silencing miR-199a-5p promoted LC cell activity, elevating miR-199a-5p did the opposite. Under rescue experiments, CDKN2B-AS1 was seen to mediate the autophagic capacity of LC cells through miR-199a-5p. This indicates that CDKN2B-AS1 and miR-199a-5p have important potential significance and research value for LC.
